# A tool for assessing the climate change mitigation and health impacts of environmental policies: the Cities Rapid Assessment Framework for Transformation (CRAFT)

**DOI:** 10.12688/wellcomeopenres.16345.2

**Published:** 2021-05-18

**Authors:** Phil Symonds, James Milner, Nahid Mohajeri, Juliette Aplin, Joanna Hale, Simon J Lloyd, Henry Fremont, Sam Younkin, Clive Shrubsole, Lawrie Robertson, Jonathon Taylor, Nici Zimmermann, Paul Wilkinson, Mike Davies

**Affiliations:** 1 UCL Institute for Environmental Design and Engineering, London, WC1H 0NN, UK; 2 Centre on Climate Change and Planetary Health & Department of Public Health, Environments and Society, London School of Hygiene and Tropical Medicine, London, UK; 3 Buro Happold Consulting, London, W1T 1PD, UK; 4 Centre for Behaviour Change, University College London, London, UK; 5 Climate and Health Program (CLIMA), Barcelona Institute for Global Health (ISGlobal), Barcelona, Spain; 6 Global Health Institute, University of Wisconsin, Madison, USA; 7 Department of Civil Engineering, Tampere University, Tampere, Finland

**Keywords:** Health impact assessment, Rapid assessment tool, Mortality, Greenhouse gas emissions, Environmental exposures, City-scale policy assessment

## Abstract

**Background:** A growing number of cities, including Greater London, have set ambitious targets, including detailed policies and implementation plans, to reach global goals on sustainability, health, and climate change. Here we present a tool for a rapid assessment of the magnitude of impact of specific policy initiatives to reach these targets. The decision-support tool simultaneously quantifies the environmental and health impacts of specified selected policies.

**Methods:** The ‘Cities Rapid Assessment Framework for Transformation (CRAFT)’ tool was applied to Greater London. CRAFT quantifies the effects of ten environmental policies on changes in (1) greenhouse gas (GHG) emissions, (2) exposures to environmental hazards, (3) travel-related physical activity, and (4) mortality (the number of attributable deaths avoided in one typical year). Publicly available data and epidemiological evidence were used to make rapid quantitative estimates of these effects based on proportional reductions in GHG emissions and environmental exposures from current baseline levels and to compute the mortality impacts.

**Results:** The CRAFT tool estimates that, of roughly 50,000 annual deaths in Greater London, the modelled hazards (PM _2.5_ (from indoor and outdoor sources), outdoor NO _2_, indoor radon, cold, overheating) and low travel-related physical activity are responsible for approximately 10,000 premature environment-related deaths. Implementing the selected polices could reduce the annual mortality number by about 20% (~1,900 deaths) by 2050. The majority of these deaths (1,700) may be avoided through increased uptake in active travel. Thus, out of ten environmental policies, the ‘active travel’ policy provides the greatest health benefit. Also, implementing the ten policies results in a GHG reduction of around 90%.

**Conclusions:** The CRAFT tool quantifies the effects of city policies on reducing GHG emissions, decreasing environmental health hazards, and improving public health. The tool has potential value for policy makers through providing quantitative estimates of health impacts to support and prioritise policy options.

## Introduction

With rapidly increasing urbanization, it is estimated that by 2050 nearly 70% of the world's population will be living in cities ( United Nations, 2018). Cities are complex systems ( Batty, 2008) and have long been known to be engines of innovation and creation of wealth. They are also key centres of pollution and diseases ( Bettencourt *et al.*, 2007). While cities provide great potential for improving our lives and well-being, there exists considerable variation in the levels of environmental exposures including (i) air pollution, (ii) noise, (iii) temperature, (iv) green space, and (v) physical activity within and between cities. This is partly due to cities having different urban-planning practices and different infrastructure systems. There is rapidly growing knowledge as to the effects of environmental exposure on human health, and this knowledge is increasingly being translated into public policy ( Caplin *et al.*, 2019).

With regard to environmental exposures, it is known that (i) air pollution, (ii) noise and (iii) temperature all have adverse health effects including increased premature mortality ( Nieuwenhuijsen *et al.*, 2017a; Nieuwenhuijsen *et al.*, 2017b). By contrast, (iv) green spaces have predominantly positive health effects ( Gascon *et al.*, 2016a; Gascon *et al.*, 2016b; Hartig *et al.*, 2014), but also some negative impacts such as contributing to urban sprawl and the spread of vector-borne diseases ( Cucca, 2012; Hartig *et al.*, 2014; Lõhmus & Balbus, 2015). As for (v) physical activity, it has many health benefits ( Warburton & Bredin, 2017). In 2008, around 31% of adults above the age of 15 (28% of men and 34% of women) worldwide had insufficient physical activity. Each year globally about 3.2 million people die because of too little physical activity ( WHO, 2014). In Barcelona, 20% of premature mortality is related to urban and transport planning-related exposures, which include air pollution, noise, temperature, available green space, and physical activity levels ( Mueller *et al.*, 2017). Many premature deaths could be prevented if recommendations for physical activity, exposure to air pollution, noise, and heat, as well as access to green space were followed.

Health impact assessment (HIA) has been recognized as one of the main tools to integrate evidence into decision-making processes, to include improved public health as a part of all policies, and to promote public health in multiple sectors ( Davenport *et al.*, 2006; IFC, 2009; Mindell *et al.*, 2004; NHS, 2002; NRC, 2011; Shafiea *et al.*, 2013; Ståhl *et al.*, 2006). The EU-funded projects INTARESE and HEIMTSA represent rare examples of integrated environmental health impact assessments ( Briggs, 2008; Hänninen *et al.*, 2014; Nieuwenhuijsen *et al.*, 2017a; Prüss-Ustün *et al.*, 2003). A combination of quantitative and participatory (citizen and stakeholder involvement) HIA tools has been used to allow policy makers to explore more accurately the positive and negative health impacts of current and future policy scenarios. However, HIAs that have been mainly conducted so far have been at the national or regional level ( Briggs *et al.*, 2016; Forouzanfar *et al.*, 2017). This indicates the importance of developing such tools for assessing health-related policies at the city scale.

Action by cities will be crucial to the fulfilment of global goals on sustainability and health, including those related to climate change. A growing number of cities have set ambitious targets for environmental action and are developing detailed policies and implementation plans to reach them. Many of these policies are expected to have substantial benefits for health and arguments about health benefits are often invoked to strengthen the case for their adoption. However, our own experience of working with city authorities suggests that, at the early stages of policy formulation, decision-makers would benefit greatly if a tool could be provided for a rapid assessment of the magnitude of impact of specific policy initiatives regarding climate change mitigation and health. We hypothesized that the provision of evidence on such joint impacts would be particularly useful to the decision process and so aimed to design a tool that would provide broad estimates of these impacts. The result was an Excel-based modelling framework known as the *Cities Rapid Assessment Framework for Transformation* (CRAFT), which we describe in this paper.

The intended features of this tool were that it should be able to provide rapid and useful, approximate quantification of both CO _2_e reduction and mortality impacts of multiple policies so that ‘order-of-magnitude’ comparisons could be made at an early stage in the decision process to help guide initial discussions of policy choices. It was not intended as a tool for detailed simulation of the effects of policies. Such detailed modelling would need to be undertaken subsequently using other methods once initial choices were narrowed. The CRAFT model was intended to provide a broad understanding of what scale of impact specific policies might be expected to achieve and with what level of change.

In this paper, we report the development and methods of the CRAFT tool (or model), illustrating its application to selected environment and health policies for Greater London, UK, in relation to transport, housing, energy, greenspace and waste. We discuss its potential strengths and weaknesses and its role as a decision-support tool and outline the process for the future evaluation of the utility of the model to policy makers and the public.

## Methods

**Figure 1.**
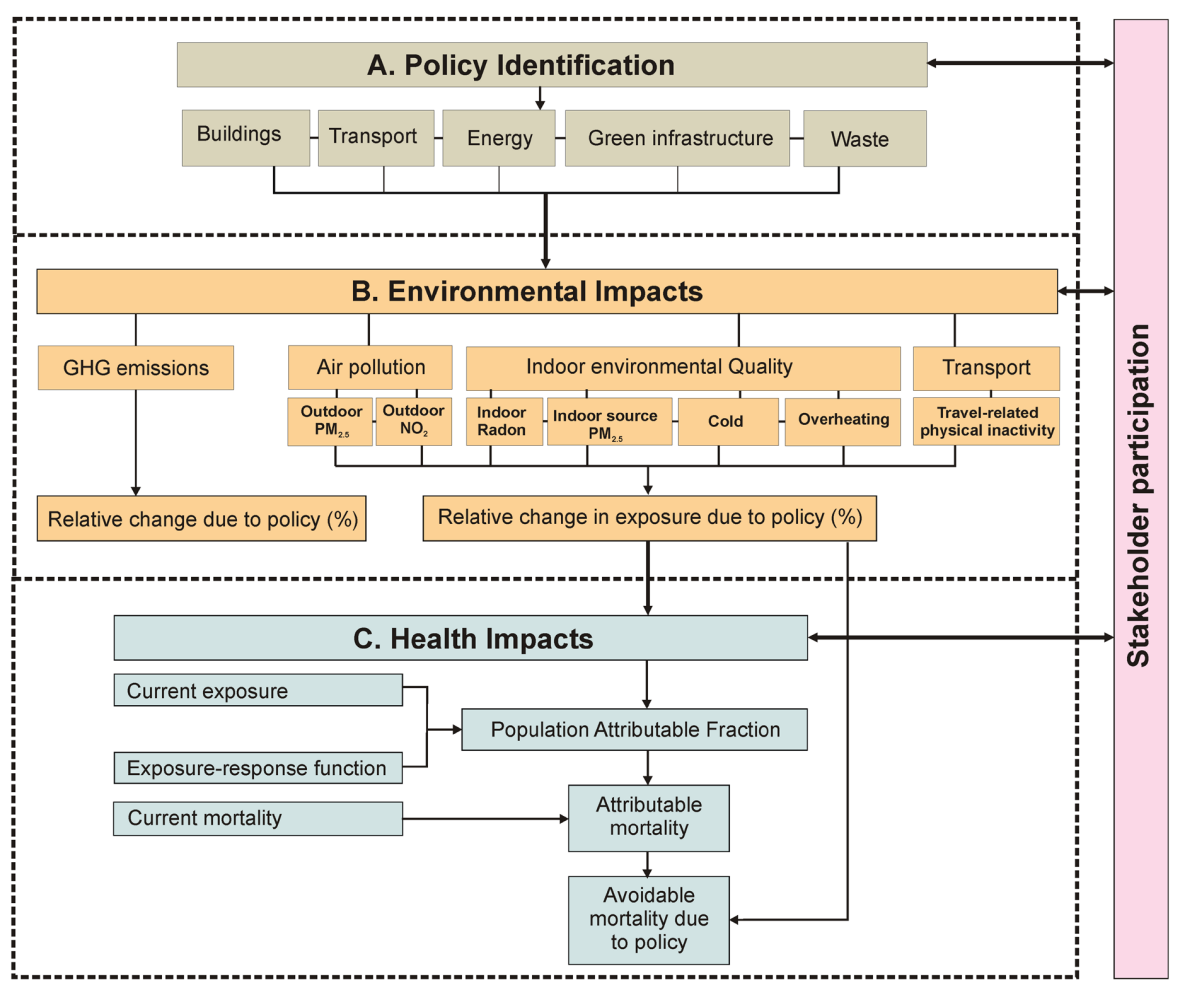
Flow diagram of the CRAFT tool. The grey colour indicates the first step of the assessment using the CRAFT tool namely, policy identification. The orange colour indicates the second step of the assessment namely, the environmental impacts. This includes GHG emissions, air pollution, and indoor air quality, as well as travel related physical inactivity. The blue colour shows the final step of the assessment, that is, the health impacts. The arrows between stakeholder participation and the assessment steps represents engagement (e.g. presenting policy choices or showing model results) and receiving feedback from stakeholders.

**Figure 2.**
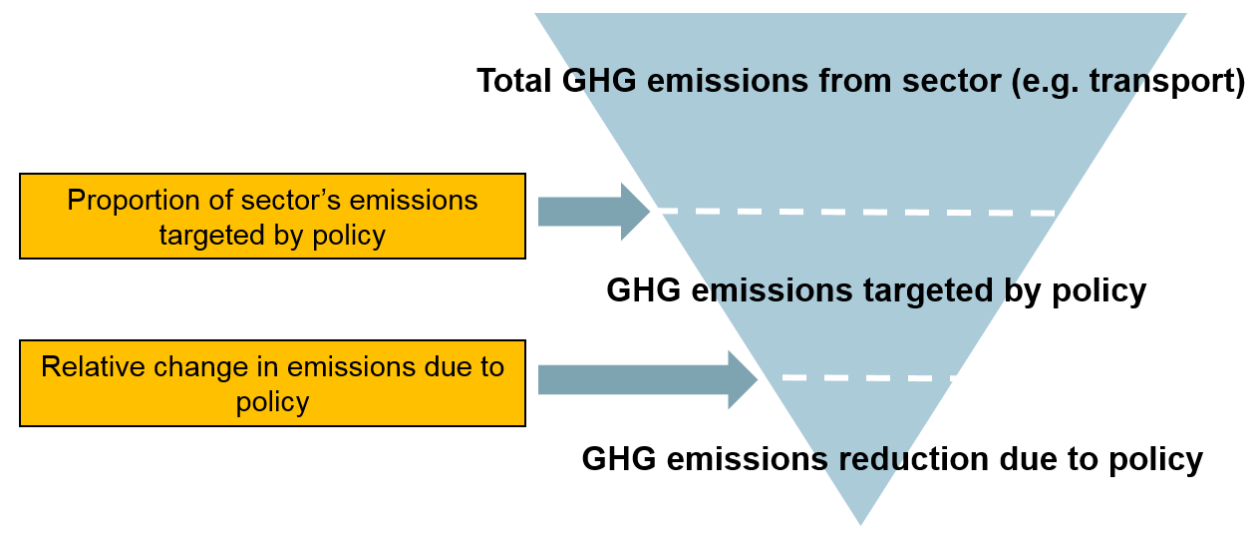
Simplified calculation steps used in CRAFT to estimate changes in GHG emissions.

The development of the CRAFT tool was undertaken as part of the research project *Complex Urban Systems for Sustainability and Health* (CUSSH), supported by the Wellcome Trust. Figure 1 provides a flow diagram of the CRAFT tool. In its current version, the tool provides estimates of the effects of specified policy choices with regard to (i) reduction in greenhouse gas (GHG) emissions in terms of tonnes of CO _2 _equivalent avoided per year and (ii) the associated change in mortality quantified as the number of attributable deaths avoided in one year. To do this, the tool uses a common principle of fractional attribution. The precise details of how this principle is applied vary somewhat from policy to policy but the general principle entails estimating the fraction of total GHG emissions that are attributable to a particular sector (transport, housing, etc.) and then to the relevant target of action within each sector. The overall impact on GHG emissions for a specific policy is then computed by multiplying relevant fractions (e.g. the fraction of emissions attributable to the target sector × the fraction of the target that will be changed as a result of the policy × the fractional reduction in emissions per unit of the target consequent to the policy action). This is more clearly understood by the example shown in Figure 2. Throughout we assumed that the linear scaling implied by this approach is a reasonable approximation to real change even though in reality there may be some non-linearities. Initial estimates of the proportion of the city’s emissions by sector, of specific sources within each sector and of the effect of the technology or behaviour change relevant to that source, were all taken from published sources, using the most relevant and up-to-date data available.

A similar process was applied for the computation of mortality impacts. The specific impacts we considered included those related to changes in (i) outdoor air pollution (particulate matter <2.5 µm (PM _2.5_), and nitrogen dioxide (NO _2_)), (ii) the indoor environment (indoor PM _2.5_, radon, cold, and overheating), and (iii) travel-related physical activity. For each selected policy, we estimated what proportion of the population might be affected by the policy and what level of change in exposure or behaviour (physical activity) of an individual within the affected population as a result of the policy. For each form of exposure or health behaviour (physical activity), the policy-modifiable attributable mortality was computed using published epidemiological exposure-response relationships (relative risks) applied to current levels of exposure (ambient air pollution, winter indoor cold, levels of physical activity, etc.) and compared with exposure as modified by the specific policy. As with GHG emissions, the calculations generally reduced to a set of fractions to estimate the change to current exposure because of the policy, from which mortality calculations were computed by applying relative risks to the exposure change in the affected proportion of the population.

In the current implementation of the CRAFT tool, we do not include calculations of morbidity impacts which, for most outcomes, are less securely estimable than for mortality and often less important if judged in terms of monetized equivalent of quality-adjusted life-years. Morbidity impacts will be added in future versions of the tool, however. For simplicity and transparency, for both GHG changes and attributable mortality, we provide calculations of the impact in one year relative to current levels of emission/exposure and assuming no time lag. In reality, there are bound to be time delays in implementation and in the evolution of impacts, sometimes substantial. However, characterizing year-by-year changes would require appreciable additional complexity and assumptions in the modelling process, which would detract from the simplicity and transparency of generating single-year estimates.

### Example application

To illustrate the use of the tool, we now describe its application to the assessment of selected environmental policies for Greater London drawn from key strategy documents published by the Mayor’s Office. Greater London, a partner city of the CUSSH project, has ambitious goals to reduce GHG emissions and to adapt to the impacts of climate change through a wide range of cross-sector actions ( Belesova *et al.*, 2018). It is also a city with a strong focus on sustainability and health equity. We chose to focus on key policy objectives outlined in the 2018 Environment Strategy for Greater London ( GLA, 2018a) and other documents, including the Mayor’s Transport Strategy ( GLA, 2018b), the Greater London Housing Strategy ( GLA, 2018c), the GLA’s Zero Carbon London: A 1.5°C Compatible Climate Action Plan ( GLA, 2018d), and the Zero Carbon Pathways (ZCP) Tool ( GLA, 2018e). We selected ten policies to model from among approximately 100 policies outlined in these documents, choosing policies that entailed relatively broad actions and which were primarily motivated by greenhouse gas emissions reduction ( Table 1).

**Table 1.** Policy ID and a short description of the selected 10 policies.

No.	Policy ID	Policy description
1	E-transport system	London's entire transport system to be zero emission by 2050 (source: London Environment Strategy)
2	Active travel	8/10 trips made on foot, by cycle or by public transport (from 6/10 today) by 2041 (source: Mayor’s Transport Strategy)
3	Buildings upgraded	Up to 50% of buildings upgraded by 2025, 100% upgraded by 2050 (source: Zero Carbon London/ZCP Tool)
4	Heat pumps	Up to two million heat pumps installed across London by 2050 (source: Zero Carbon London/ZCP Tool)
5	Heat networks	Tenfold increase in heat networks by 2025, connecting up to 650,000 homes to waste and environmental heat sources by 2050 (source: Zero Carbon London/ZCP Tool)
6	PV installations	Up to 100,000 photovoltaic installations across London by 2025, increasing to 25% of all viable buildings by 2050 (source: Zero Carbon London/ZCP Tool)
7	Grid decarbonisation	Grid decarbonisation in line with UK carbon budgets. High penetration of renewables and nuclear, doubling capacity by 2030 (source: Zero Carbon London/ZCP Tool)
8	Green gas supply	Green gas in national supply increasing significantly from 2030, contributing 13% of gas supply by 2050 (source: Zero Carbon London/ZCP Tool)
9	Greenspace	Increase London's green area from 47% to >50% by 2050 (source: London Environment Strategy)
10	Zero waste city	London will be a zero waste city. By 2026 no biodegradable or recyclable waste will be sent to landfill, and by 2030 65 per cent of London’s municipal waste will be recycled. (source: London Environment Strategy)

In this section, we describe the data sources and the key assumptions that are made to estimate the changes in GHG emissions and mortality impact of changes to environmental exposures and physical activity associated with the policy actions.

### GHG emissions

**Figure 3.**
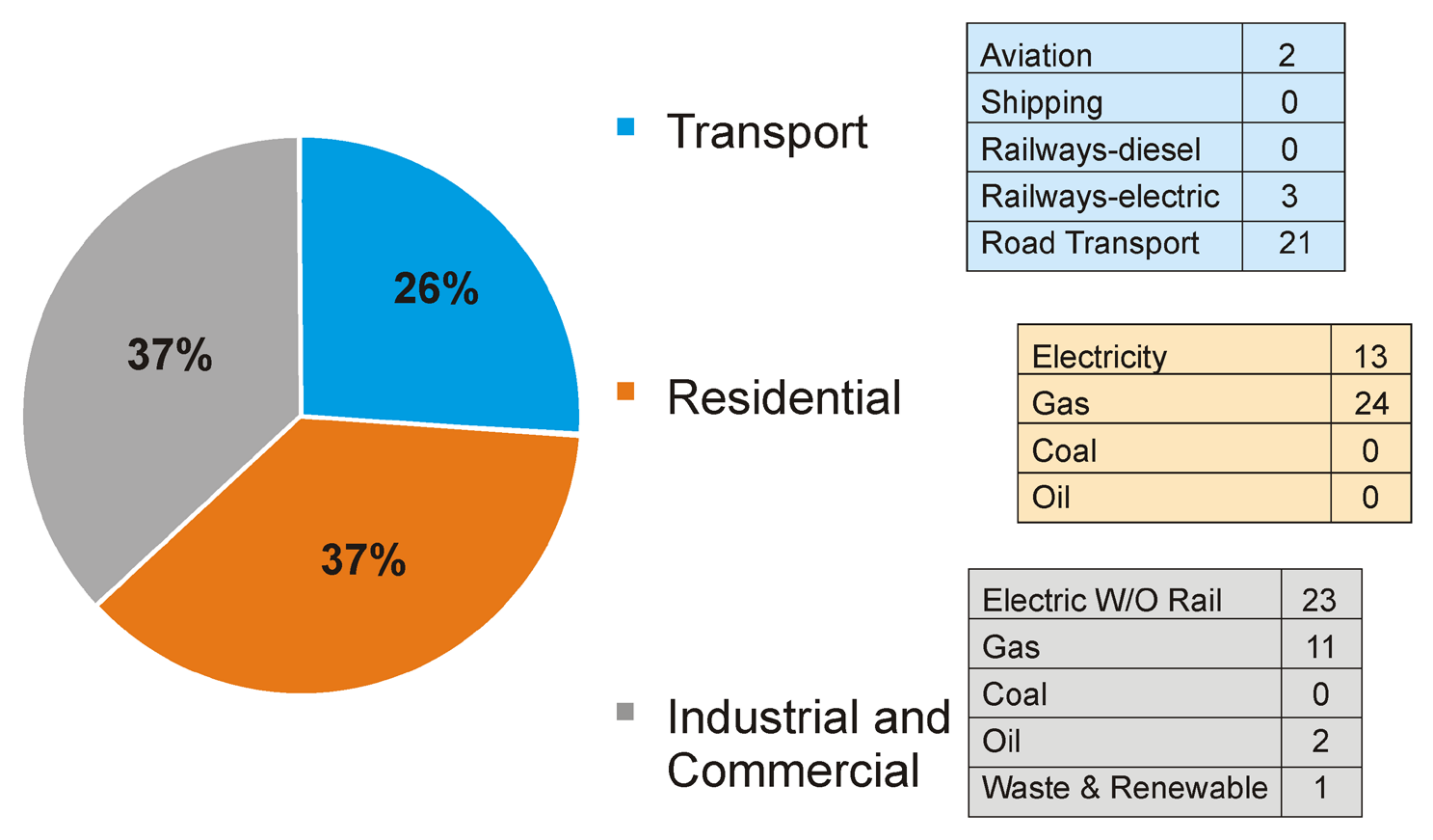
Greater London GHG emissions by sector in % include both direct (Scope 1) and indirect (Scope 2) GHG emissions [reproduced with permission from LEGGI; GLA, 2016a)].

Our calculation for Greater London’s GHG emissions were based on Scope 1 & 2 emissions as defined by the Greenhouse Gas Protocol ( GLA, 2016a), namely all direct GHG emissions produced within the city’s boundary (Scope 1) and indirect GHG emissions from consumption of imported electricity, heat or steam (Scope 2). We did not include Scope 3 emissions, i.e. those associated with manufactured and purchased goods. It is important to note, however, that at 115 MtCO _2_e, Greater London’s Scope 3 emissions are estimated to account for more than three times Greater London’s direct emissions of around 34 MtCO _2_e. Estimates of the sector contributions to Greater London’s Scope 1 & 2 GHG emissions were derived from *The London Energy and Greenhouse Gas Inventory (LEGGI)* ( GLA, 2016a) and are summarized in Figure 3.

The GHG emissions associated with each of the ten policies was estimated using the principle of 'fractional attribution’ outlined above, i.e. by the product of the fractions reflecting the proportion of overall GHG emissions attributable to the sector, the proportion of those sectoral emissions targeted by the policy and the proportional reduction in emissions from those targeted. The data sources and key assumptions used in these calculations are summarized in Table 2.

**Table 2.** Data source and the assumptions used for GHG emissions calculations.

NO.	Policy ID	Sources	Assumptions
1	E-transport system	( BEIS, 2018)	Assume entire transport system is electrified. Use the BEIS Green book emissions factor is used which suggest that GHG emissions from the grid will reduce by ~95% from 2010 levels by 2050.
2	Active travel	London Travel Demand Survey ( TFL, 2018); ( EEA, 2015)	The current mode share from the TFL’s Travel Demand Survey is used. These mode shares are converted to typical distances travelled and then multiplied by the CO _2_ per passenger kms from EEA (2015).
3	Buildings upgraded	Hamilton *et al.*, 2015	Predictions for changes in energy savings due to energy efficiency retrofit are used
4	Heat pumps	( BEIS, 2018)	Assume electrification of heat and decarbonisation of the grid in line with BEIS Green book. Also assume a coefficient of performance (COP) of 3 for heat pumps.
5	Heat networks	DECC, 2009	Take assumptions on CO _2_e savings from heat networks compared to conventional heating systems from a report commissioned by DECC
6	PV installations	( BEIS, 2018); GLA, 2018f	The ratio of energy demand that could be met by solar was taken from the GLA’s Solar action plan. BEIS Green book emission factors were again applied.
7	Grid decarbonisation	( BEIS, 2018)	Total electricity demand GHG emissions (LEGGI) and BEIS Green book emission factors were used.
8	Green gas supply	NA	No additional data requirements.
9	Greenspace	GLA, 2015	Carbon Sequestration from the urban forest and energy savings from buildings reported in Valuing London's Urban Forest ( GLA, 2015) were used.
10	Zero waste city	GLA, 2017	The reduction in CO2e from waste management used was taken from ‘Greenhouse Gas Emissions Performance Standard for London’s Local Authority Collected Waste – 2017 Update’ ( GLA, 2017).

### Mortality impact of change to environmental exposures

The method used to estimate changes in mortality related to environmental exposures and health-related behaviours as a result of each policy is broadly similar to that used for the GHG emissions. Namely, we calculate the proportional contributions from the relevant sector, the sources targeted by the policy within the sector, and then the relative change achieved among the targeted sources by the specific action (i.e. by the policy-related intervention). Baseline mortality rates for the Greater London population were based on data from the Office for National Statistics for the year 2017.

**Figure 4.**
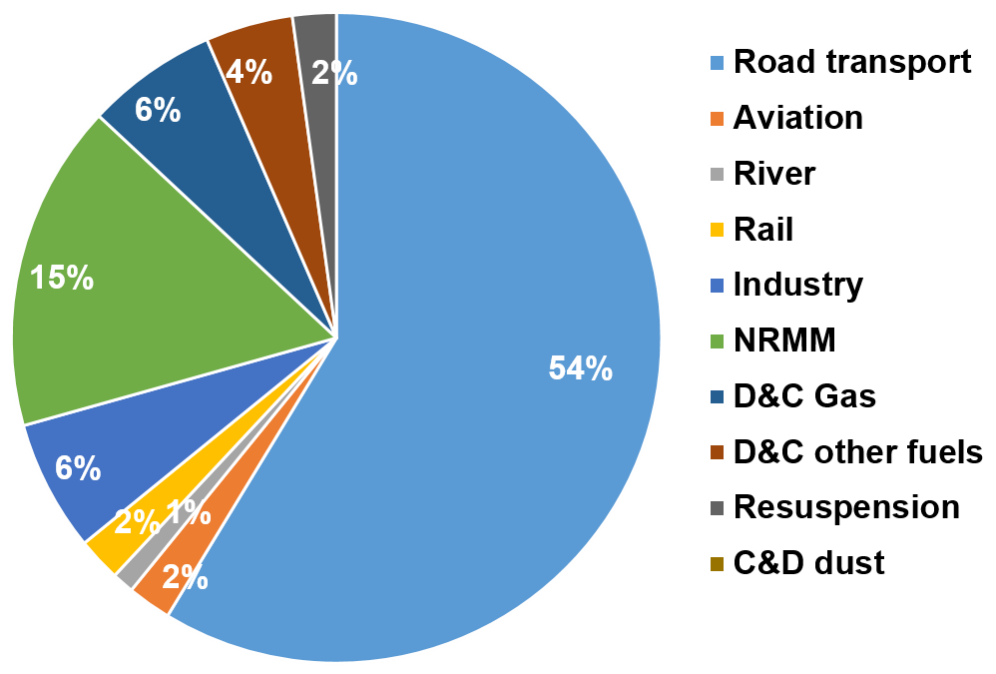
Greater London outdoor particulate matter, PM _2.5_ emissions by sector [reproduced with permission from LAEI ( GLA, 2016b)].

For outdoor air pollution, we assumed that 25% of ambient (background) PM _2.5_ and 80% of the NO _2 _concentrations in Greater London are due to emissions within the GLA ( Howard, 2015). The changes in PM _2.5_ and NO _2_ concentrations from policies were then assumed to occur in proportion to the change in local source emissions for these pollutants. The proportion of emissions produced within GLA by sector and transport type were obtained from the Greater London Atmospheric Emissions Inventory (LAEI) ( GLA, 2016b) ( Figure 4). Our primary calculations of outdoor air pollution-related mortality were based on changes to PM _2.5_ alone, using an exposure-response coefficient derived from ( Pope *et al.*, 2002). However, because of increasing evidence of independent effects on mortality of NO _2_, we also carried out parallel calculations for change in NO _2_ concentrations using exposure-response functions derived from ( COMEAP, 2018). The impacts based on these calculations are not independent and should not be summed. They were included as alternatives to show the different estimates derived from these methods, especially for transport-related policies, which have much greater impact on NO _2_ emissions and concentrations than on those for PM _2.5_.

For changes to the indoor environment, we used methods of health impact calculation outlined in Hamilton *et al*. (2015). for cold, heat ( Taylor *et al.*, 2018), indoor PM _2.5_ and radon. A key uncertainty with these calculations was the change in ventilation characteristics of the dwelling consequent to home energy-efficiency interventions. Depending on the assumption about changes in ventilation following retrofit, the net impacts on mortality can be negative or positive. Additional data sources along with key assumptions used in the estimations are listed in Table 3.

**Table 3.** Data source and the assumptions used for environmental exposure calculations.

NO.	Policy ID	Sources	Assumptions
1	E-transport system	NA	No additional data requirements.
2	Active travel	London Travel Demand Survey ( TFL, 2018), ( BEIS, 2012)	As with GHG emissions, the London Travel Demand Survey is used to establish the current proportion of passenger kms travelled by various modes. Transport emission factors for various pollutants per km were obtained from the National Atmospheric Emissions Inventory ( BEIS, 2012).
3	Building upgraded	Hamilton *et al*., 2015; Taylor *et al*., 2018	Predictions for changes in indoor environmental exposures due to home energy efficiency retrofit were taken from Hamilton *et al*., 2015. This includes indoor and outdoor PM _2.5_, Radon, mould and standard indoor temperature. Predicted changes in overheating were based on work by Taylor *et al*. (2018).
4	Heat pumps	NA	No additional data requirements. Heat pumps assumed to completely eliminate direct emissions of pollutants from homes.
5	Heat networks	NA	No additional data requirements. Heat networks assumed to eliminate direct emissions of pollutants.
6	PV installation	DEFRA, 2019	Emissions due to power generation were determined from DEFRA (2019). This was multiplied by the reduction in demand due to the adding solar PV.
7	Grid decarbonisation	DEFRA, 2019	Emissions due to power generation were determined from DEFRA (2019). It is assumed grid decarbonisation would eliminate the majority of these emissions.
8	Green gas supply	NA	No data currently available.
9	Greenspace	GLA, 2015	Proportion of pollution removed by the urban forest was obtained from Valuing London's Urban Forest ( GLA, 2015).
10	Zero waste city	NA	No data currently available.

For transport-related physical activity, we assessed changes in transport-related metabolic equivalent of the task (MET) hours per week. The MET for a specific task is defined as the ratio of the rate of energy consumption by that person while performing that activity compared with a reference. By convention the reference is taken as 3.5 mL of oxygen per kilogramme body mass per minute, which is approximately the energy expended when sitting at rest ( Jetté *et al.*, 1990). Data from the London Travel Demand Survey ( TFL, 2018) was used to estimate the current mean level of travel-related physical activity in Greater London (7.65 MET-hrs/week). The mean level of leisure related physical activity (not including travel activity) was estimated (11.25 MET-hrs/week) using data from Arem *et al.* (2015). Changes to all-cause mortality risk were then based on estimates of change to the sum of these two sources of physical activity, applying a piecewise linear exposure-response function for all-cause mortality, constructed with data from Arem *et al.* (2015).

For this version of CRAFT the mortality impact associated with changes in green space was based only on calculations of the estimated effect of green space on ambient air pollution – specifically the proportion of pollution removed by the urban forest obtained from the 2015 report *Valuing London's Urban Forest* ( GLA, 2015). This omits any effects associated with, for example, protection against heat risk in summer, increased levels of physical activity, improved mental well-being and other effects that depend on the form of green space change and for which the methods of quantification remains uncertain.

### Policy interactions

Where possible, the CRAFT tool attempts to account for the interactions between policies. We consider two general types of interaction. First, dependencies, where the benefits achieved by a given policy are contingent on the degree of implementation of at least one other policy. Second, double-counting, in which a given impact is (potentially) achieved independently by more than one policy. An example is the effect of the interaction between ‘E-transport system’ (policy 1) and *‘*Active travel’ (policy 2), on GHG emissions which is mediated by a third policy namely, ‘Grid decarbonisation’ (policy 7). The first step is to determine which policy will be implemented first chronologically. In this case, policy 2 has a target of the year 2041, while policy 1 has a target of the year 2050. Therefore, reduced demand in non-active travel types due to modal shift is subtracted from policy 1. Policy 1 is also contingent on a third policy, ‘Grid decarbonisation’ being fully achieved. If grid decarbonisation is not achieved, the GHG reducing benefits of zero emission transport decrease because it is assumed to be electric powered. As indicated above, the possibility and the nature of any interactions depends on the assumptions embedded in the initial calculation of the impacts of a given policy.

## Results

**Figure 5.**
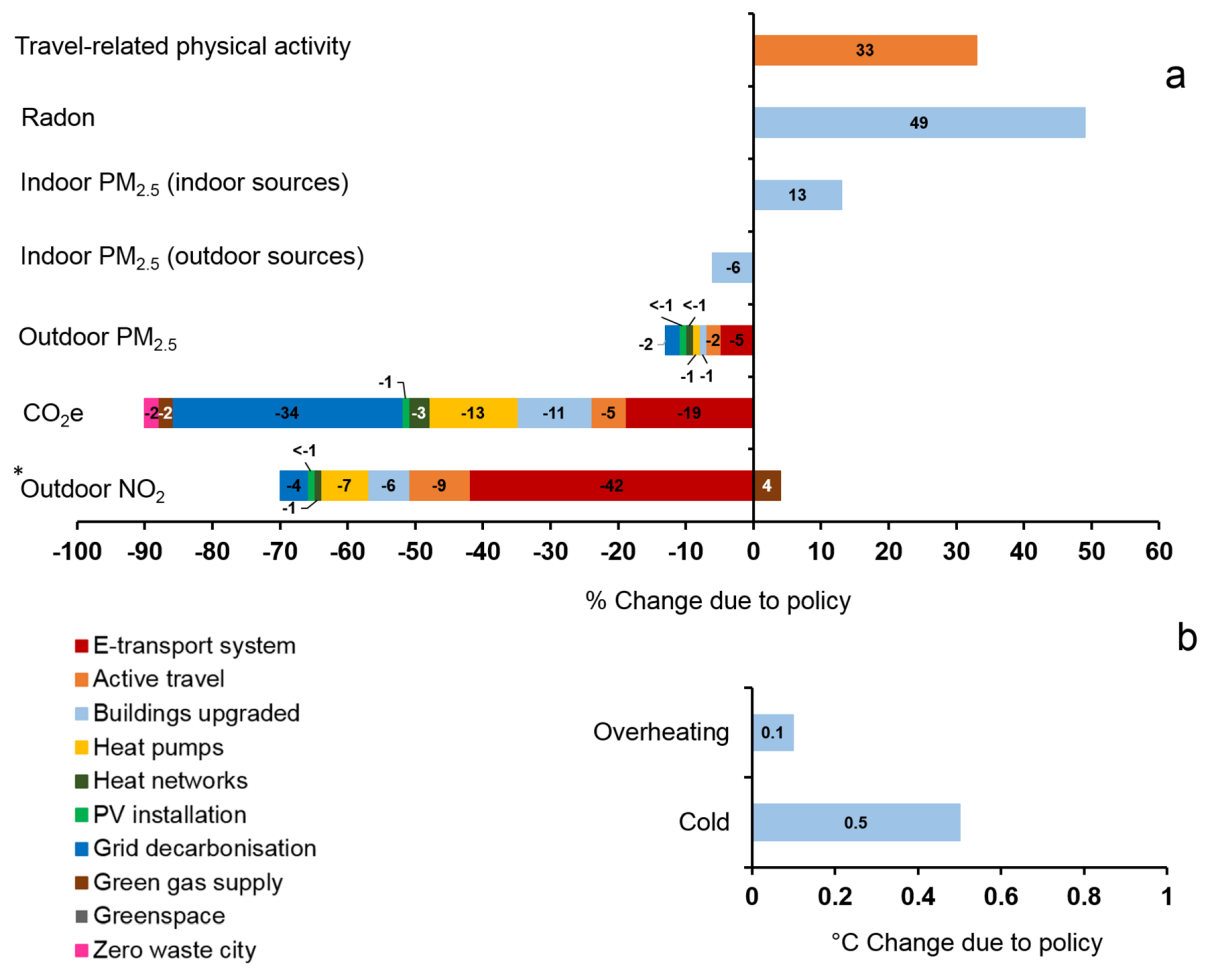
( **a**) Central estimates of percentage changes in GHGs emissions (i.e. CO _2_e), air pollution (outdoor PM _2.5_, outdoor NO _2_), indoor environmental quality (indoor radon, indoor PM _2.5 _(indoor sources), indoor PM _2.5 _(outdoor sources)), and transport (travel-related physical activity) due to the 10 policy actions in London (policy IDs are shown in colour). Left-hand side represent decrease (values shown in minus) and right-hand side represent increase in % changes due to policy. ( **b**) Central estimates of absolute degree changes of ‘Overheating’ and ‘Cold’ due to one policy action in London. *Please see Figure 6.

**Figure 6.**
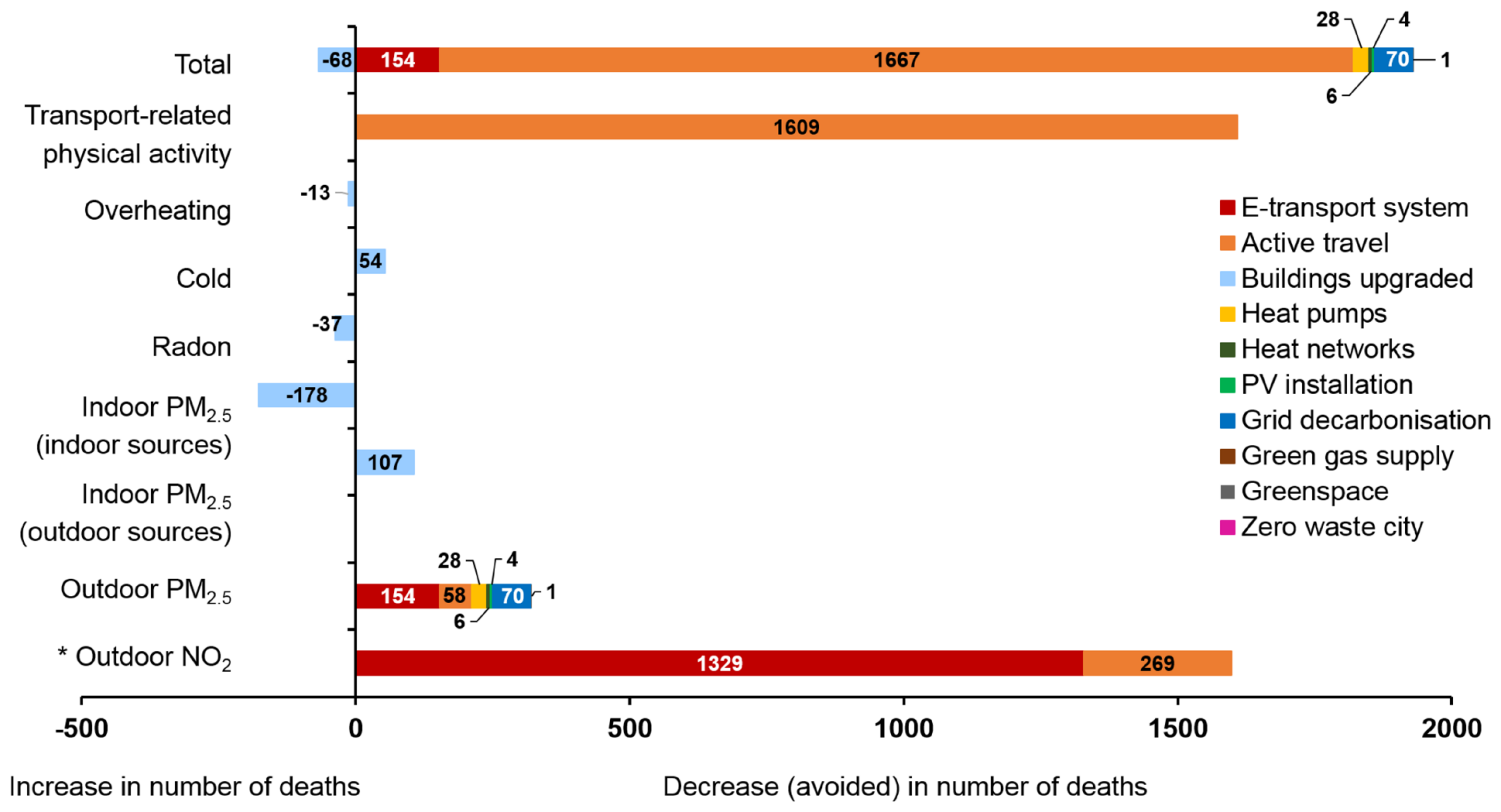
Estimated impact on health due to the 10 key policy actions in London (the policy IDs are shown in colour). * Notice that the impacts for NO _2_ has not been added to the PM _2.5_ and thus to the total. We show the NO _2_ results alongside those for PM _2.5_ for completeness and to indicate the uncertainties involved. On the right-hand side is the decrease in number of deaths (avoided deaths) and on the left-hand side the increase in number of deaths due to 10 key policy actions.

The results for the ten policies assessed by the CRAFT tool are shown in Figure 5 (central estimates of percentage changes in GHGs emissions and environmental exposures) and Figure 6 (estimated impact on health due to the 10 key policy actions in Greater London). *Extended data*, Supplementary File 1 ( Symonds *et al.*, 2020a) presents the detailed calculation steps for each policy, along with assumptions used for current and minimum exposures and the exposure-mortality coefficients and population mortality statistics used. Overall, fully implementing all ten selected policies could reduce the environmental disease burden in Greater London by about 1,900 deaths in one year, or around 20% of current mortality. This total is based on calculations in which the impact of outdoor air pollution is based on changes to PM _2.5_ only (to avoid potential double counting due to outdoor PM _2.5_ and NO _2_). In terms of reduction to CO _2_e emissions, the largest contribution would occur from grid decarbonization, followed by the ‘zero emission’ transport system, use of heat pumps and buildings energy efficiency upgrades ( Figure 5).

Most policies were estimated to result in a reduction of harmful exposures. The transport related policies (‘E-transport system’ and ‘Active travel’) have the greatest potential for reduction of both CO _2_e emissions and mortality. By far the largest contributor to reducing mortality was estimated to be active travel, with a population impact for the increase in active travel greater than all other changes in exposure combined. However, if the calculation of mortality following changes to outdoor air pollution were based on NO _2_ rather than PM _2.5_, these impacts would be only a little lower than those for physical activity ( Figure 6).

The results relating to housing energy efficiency interventions (‘Buildings upgraded’) are mixed, partly because of the uncertainties over changes to ventilation characteristics. The ‘buildings upgraded’ policy has the potential to lead to worse indoor air quality in homes in Greater London if energy efficiency is not supported by provision of adequate compensatory ventilation. In the assumptions made for this analysis, the reduction in mortality from lower outdoor PM _2.5_ are more than offset by home energy efficiency-related increases in indoor PM _2.5_ derived from indoor sources. Radon-related mortality was also estimated to increase because of the reduced ventilation to homes. Overall, heat related mortality was also estimated to increase because of the nature of the interventions. However, the reduction in deaths due to cold was estimated to be appreciably larger than the increase in heat-related mortality. Energy efficiency installations can be net beneficial for health if ventilation is protected and steps taken to address overheating. Installation of two million heat pumps for homes (‘Heat-pump’ policy) would reduce Greater London’s GHG emissions substantially (contingent on grid decarbonisation) but provide relatively modest benefits for health by reducing ambient air pollution. The ‘Heat networks’ and ‘Green gas supply’ policies would have more limited GHG reduction and health benefits.

Our estimates of the impact of increasing greenspace in Greater London from 47% to 50% (the ‘Greenspace’ policy) would have only relatively minor impact on GHG emissions and mortality, given the limited pathways to health included in the calculations. Making Greater London a zero-waste city would reduce GHG emissions by about 1–2%. Overall, the ten policies reduce outdoor PM _2.5_ concentrations in Greater London by around 12% (excluding the effects of policy 3 on indoor exposures), which would reduce annual PM _2.5_-attributable premature mortality by around 300 deaths. *Extended data*, Supplementary File 2 ( Symonds *et al.*, 2020b) shows the results for each policy separately, ignoring any potential interactions.

## Discussion

The CRAFT tool is designed to be a decision-support tool that provides approximate, initial comparison of policy choices in terms of changes to GHG emissions and mortality. Its results are based on approximate methods of calculation and are not intended to be a substitute for more detailed modelling that may be needed to examine specific policy choices in depth. However, its relatively approximate results do allow a quick comparison of multiple policy choices in terms of CO _2_e and mortality impacts, and thus have a potential role in the early phase of policy development. The results for our first analysis of selected environmental policies for Greater London show that there are large differences between major policy choices and that in some cases (as for housing) also important uncertainties about the health impacts depending on the detail of the policy implementation.

How valuable the analysis is to decision-makers remains to be determined in future evaluations. However, the key strengths of the CRAFT tool appear to be assessing the health impacts of city policies rapidly at an early stage to support the decision-making process and to help prioritise different actions. Furthermore, the tool allows exploration of how policies can be modified so as to provide opportunities for increasing physical activities and mitigation of environmental hazards. The tool, however, has a number of limitations, specifically as regards validation and uncertainty assessment. Uncertainties arise at every stage in the analysis from the initial issue framing, through data collection, assumptions, and modelling to the interpretation and reporting of the results. In a rapid assessment such as this, detailed quantification of uncertainties is not possible. Complex dynamic models have the capability to assess the uncertainties in much greater detail.

Using the CRAFT tool, we estimate the percentage changes in GHGs emissions, air pollution (outdoor PM _2.5_, outdoor NO _2_), indoor environmental quality (indoor radon, indoor PM _2.5 _(indoor source), indoor PM _2.5 _(outdoor source), and transport behaviours (travel-related physical activity) that may be achieved by ten key environmental policy actions in Greater London. Of these policies, the highest reduction in GHG emissions is 34% due to the policy action ‘Grid decarbonisation’. The lowest reduction, less than 1%, is due to the policy ‘Greenspace’. Implementing all ten policies results in a GHG reduction of around 90%, which is broadly in line with that projected in Greater London’s zero carbon plan ( GLA, 2018d). It should be noted that these proportional reduction in emissions do not account for GHG emissions related to the manufacturing and transport of imported goods or due to projected population rises.

Regarding air pollution, the highest reduction in PM _2.5 _is 5% and in NO _2_ is 42% both due to the policy action ‘E-transport system’. Increasing the proportion of trips made by foot, cycle or public transport (‘Active travel’) reduces CO _2_ emissions by 5%, PM _2.5 _by 2%, and NO _2_ by 9% and increases the level of transport related physical activity by 33%. This would have considerable benefit for public health, with about 1,700 annual premature deaths avoided, primarily through increased levels of transport-related physical activity.

In this study, health impact calculations have been performed for mortality only and therefore represent underestimates of the full positive impacts on health and quality of life that may be achieved. In some cases, impacts on morbidity may add appreciably to those on mortality; for example, recent evidence has suggested an effect of NO _2_ on asthma incidence in children ( Achakulwisut *et al.*, 2019; Bergmann *et al.*, 2020). There may also be additional environmental hazards and pathways to health which we have not identified or been able to quantify. Nonetheless, we believe that the estimates we have generated indicate the main health impacts of different policies and broadly their relative scales.

Our method estimates the theoretical number of premature deaths per year attributable to each exposure at present levels and under an alternative scenario in which the policy has been implemented in full. However, the maximum health benefit achieved may be delayed in time due to a number of factors. This includes the rate of policy implementation and uptake (for example, how quickly the policy reduces the exposure or changes behaviours) and lag times between reductions in exposure and reductions in health risk. It is therefore not appropriate to add or multiply the annual estimates to derive estimates over longer time periods. These issues can be investigated in greater depth using more sophisticated modelling tools (including data-driven modelling) being developed through the CUSSH project. For example, a microsimulation model, MicroEnv, developed recently aimed at quantifying the impact of environmental exposures on health, outputs temporal health impacts at a high spatial resolution ( Symonds *et al.*, 2019a).

In the current implementation of the tool, we have estimated health effects resulting from exposure to NO _2_ for transport policy actions only. The methods for NO _2_ health impact calculation remain uncertain and somewhat controversial. NO _2_ and PM _2.5_ tend to be highly correlated and it is difficult to separate their effects. Even from mutually adjusted two-pollutant models, it is not clear how much of apparent NO _2_ effect is causally independent of PM: PM _2.5_ coefficients probably capture some, though not all, of the NO _2_ effects, and vice versa ( COMEAP, 2018). The more established method of mortality calculation remains that based on PM _2.5_, and it is this that we present as the main analysis. The NO _2_ results are shown for comparison only and cannot be added to those of PM _2.5_. The NO _2_ results are shown to indicate an alternative method of computation in which NO _2_ is being used as an indicator of NO _2_ and the pool of other causal pollutants with which it is correlated. We show the NO _2_ results alongside those for PM _2.5_ for completeness and to indicate the uncertainties involved. NO _2_-based calculations tend to give contrasting results for transport interventions because of the relative importance of transport sector emissions to ambient NO _2_ concentrations.

 As can be seen, estimates of the health impact of transport-related interventions are generally much larger when based on NO _2_ rather than PM _2.5_, which is important for consideration of actions aimed at the transport sector. It still remains that estimates based on PM _2.5_ are more widely accepted among epidemiologists and are the ones that we suggest as the principal estimates of air pollution-related health impact.

The fact that our calculations of the impact of green space were based only on estimates of change in ambient air quality is a limitation and may mean appreciable under-estimation of the potential benefits of interventions that increase green space. The possible connections between green space and health are multiple and complex but depend on the type and quality of green space and where it is implemented ( Houlden *et al.*, 2018; Markevych *et al.*, 2017). A recent review ( Twohig-Bennett & Jones, 2018) suggests that mortality is lower in areas with greater green space, and there is evidence that inequalities in mortality risk are lower in greener areas ( Mitchell & Popham, 2008). However, it is far from certain that these associations can be translated into mortality benefits by intervention, especially when the form of new green space is not specified. It is possible that the proposed changes in green space in Greater London (as defined in terms of a satellite view) will have only a marginal effect on meaningful exposures for mortality that are not accounted for through other pathways (e.g. travel behaviour). But there could be an appreciable effect that we do not represent in the tabulations we present in this study.

The calculations for the ‘Buildings upgraded’ policy ( *Up to 50% of buildings upgraded by 2025, 100% upgraded by 2050*), see *Extended data*, Supplementary Files 1 and 2 ( Symonds *et al.*, 2020a; Symonds *et al.*, 2020b), for full details, represent our central estimate of the effects of home energy efficiency upgrades on health. Recent evidence regarding the provision of ventilation following retrofitting suggests that, if current practice continues, energy efficiency upgrades will most likely reduce levels of home ventilation and increase exposures to pollutants generated inside the home, as well as increasing over-heating related deaths ( Shrubsole, *et al.*, 2015; Symonds, *et al.*, 2019b). Although there will be reduced exposures to outdoor-generated air pollution and cold-related deaths, our estimates suggest the net impact on public health will be negative. A more substantial health benefit may be achieved if home energy efficiency measures are installed alongside ventilation that complies with UK building regulations, which would result in a net positive effect on health overall. Properly implemented, such measures can also provide increased protection against heat.

In this study, we focus on the mortality burden associated with exposures related to ten environmental policies in Greater London. There are, however, several other exposures that can be explored and their health impacts quantified in future work. This includes, quantifying road traffic injures/deaths resulting from transport mode, noise (outdoor and indoor activity noise), and effects on mortality and morbidity relating to access to green space. Changes in green space in particular are likely to have appreciable effects additional to those quantified in this report that were confined to air quality changes alone. There are however challenges in doing such quantification, which ideally, needs to take account of the specific forms of green space change and a range of different health effects.

## Conclusion

We have developed a new rapid assessment tool (CRAFT), *Extended data*, Supplementary File 3 ( Symonds *et al.*, 2020c), to quantify the effects of city policies on reducing GHG emissions, decreasing environmental health hazards and improving public health. With relatively simple assumptions and methods, the model can show appreciable differences in expected impact of different policy options. The tool has potential value for policy makers through providing quantitative estimates of health impacts to support and prioritise policy options, especially for the early phase of policy development. The assessment of the tool’s utility to decision-makers will be an important next step. That assessment should consider how the policies to be compared are selected, the form of evidence presentation, the stakeholder groups involved in the process and the processes of (iterative) engagements with them.

## Data availability

### Underlying data

All data underlying the results are available as part of the article and no additional source data are required.

### Extended data

Figshare: A tool for assessing the climate change mitigation and health impacts of environmental policies. https://doi.org/10.6084/m9.figshare.13011533.v3 ( Symonds *et al.*, 2020a).

Supplementary File 1. This file contains assumptions; calculation steps; central estimates of percentage changes in GHGs emissions, indoor and outdoor air pollutants, housing-related risks, and travel-related physical activity due to ten key policy actions in Greater London.

Figshare: A tool for assessing the climate change mitigation and health impacts of environmental policies. https://doi.org/10.6084/m9.figshare.13011539.v4 ( Symonds *et al.*, 2020b)

Supplementary File 2. This file contains estimated impact on health (i.e. theoretical premature number of deaths avoided in one year) and CO _2_ emissions due to ten key policy actions in Greater London without interactions between policies.

Figshare: A tool for assessing the climate change mitigation and health impacts of environmental policies. https://doi.org/10.6084/m9.figshare.13125407.v3 ( Symonds *et al.*, 2020c).

Supplementary File 3. The file contains a ‘Cities Rapid Assessment Framework for Transformation (CRAFT)’ tool (Excel format) which quantifies the effects of environmental policies on changes in (1) greenhouse gas (GHG) emissions, (2) exposures to environmental hazards, (3) travel-related physical activity, and (4) mortality (the number of attributable deaths avoided in one typical year).

Extended data are available under the terms of the Creative Commons Attribution 4.0 International license (CC-BY 4.0).
