# Supplementary material for: A tool for assessing the climate change mitigation and health impacts of environmental policies: the Cities Rapid Assessment Framework for Transformation (CRAFT)
Source: Wellcome Open Res. 2021 May 18;5:269. Originally published 2020 Nov 12. [Version 2] doi: 10.12688/wellcomeopenres.16345.2 (PMC8280949; doi:10.12688/wellcomeopenres.16345.2)
Supplement: Peer review history

# Peer review: Reviewer response for version 2

**DOI:** 10.21956/wellcomeopenres.18616.r44920

**Published:** 2021-07-28

**Authors:** Rebecca K. Saari

**Affiliations:** 1 Department of Civil and Environmental Engineering, University of Waterloo, Waterloo, Canada

The myriad health-related benefits of GHG reductions are known to be significant. However, they are seldom integrated into policy decisions or designs, especially at the early screening phase. To address this, decision support tools at the city scale are critically needed. I commend the authors on their response to this challenge. They present an accessible, credible tool that helps to integrate these benefits into early decision processes. The methods are appropriate for the order-of-magnitude screening they aim to provide.

 Following a careful technical review of the tool, I find the approaches defensible and grounded in solid data and research. They are well documented in the supplemental materials. However, I did wish that some of the key assumptions were better described in the manuscript itself to aid understanding and interpretation of the results. I have highlighted a few key assumptions that can affect the sign of the impact, or the magnitude of the largest impacts. I would encourage the authors to consider minor edits based on my specific suggestions below.

**Comments on assumptions and details for specific methods:**

Active Travel:

 More detail in this paragraph “For transport-related physical activity, we assessed changes in transport-related metabolic equivalent of the task (MET) hours per week… ” would be helpful. The authors cite the current METs. I recommend citing the METs under the policy so the magnitude of the change is clear, and it’s evident that only travel-related MET increases (as expected). The current wording “based on … changes in the sum of these two sources of physical activity” implies that leisure related physical activity is also affected by policy.

 I am aware that this is a particular detail that will not affect the conclusions of this work, but, for the sake of reproducibility, I also recommend describing the piecewise linear RR developed. The authors mentioned using a piece-wise linear RR developed from Arem et al. (2015), but, unless I am missing something, appear not to have described why or how they did so. They cite an RR of 0.75 for a MET of 11.25, yet Arem et al (2015) provides a piece-wise figure of hazard ratios, citing 0.69 for an MET range of 7.5-15. The authors have apparently performed a linear interpolation within the categories that goes beyond the original epidemiologic evidence, the results of which depend on how the categories are represented.

Building Upgraded:

 I recommend, in the Methods text or Table 3, describing the particular assumptions used here earlier on, in addition to mentioning that, “Depending on the assumption about changes in ventilation following retrofit, the net impacts on mortality can be negative or positive.” Otherwise, it is only in the final paragraphs of the Discussion (or in a footnote of the supplemental material) that readers learn that this reflects the “Installer Discretion” Scenario from Hamilton et al. (2015). This work also expands on Hamilton et al (2015) by including changes in outdoor air quality, another detail that could be mentioned, e.g., in Table 3, to better clarify the relation of this work to its sources.

Zero Waste City:

 As a carbon policy, the literature is clear that zero waste / circular economy policies are valuable because they reduce carbon-intensive production of goods to meet demand. Scope 1 and 2 emissions associated with city-scale waste treatment are minor compared to the embodied carbon associated with the consumption driving that waste. The authors allude to this when mentioning the primacy of London’s Scope 3 emissions. I recommend that they also explicitly discuss how excluding these emissions can undervalue zero waste policies. While the system boundary chosen in CRAFT is clear and defensible, it will dictate the relative attractiveness of this option to decision makers.

**Other Specific comments:**

 “what level of change in exposure or behaviour (physical activity) of an individual within the affected population as a result of the policy.” This phrase seems to be missing a verb since “change” and result” are both used here as nouns… suggest replacing “as a result of” with “would result from”

Figure 1:

 Can you please, when introducing Figure 1, clarify whether this is the ideal or actual flow diagram for the tool as described in this manuscript? The Figure suggests that stakeholders were involved throughout, but conclusions indicate that stakeholder participation is a next step – an apparent contradiction. 

 I found this wording confusing: “The overall impact on GHG emissions for a specific policy is then computed by multiplying relevant fractions (e.g. the fraction of emissions attributable to the target sector × the fraction of the target that will be changed as a result of the policy × the fractional reduction in emissions per unit of the target consequent to the policy action).” There appears to be a mid-sentence change in referring to a “target sector” and the “target of action”, but “target consequent to the policy action” is ambiguous and could still refer to a target sector. Possible alternative: replace “the fractional reduction in emissions per unit of the target consequent to the policy action” with “fraction of emissions reduced per unit change in the target of action”.

Results

*“Extended data*, Supplementary File 1 ( Symonds *et al.*, 2020a) presents the detailed calculation steps for each policy, along with assumptions used for current and minimum exposures and the exposure-mortality coefficients and population mortality statistics used.”

 Please introduce this early in the Methods section, e.g., near, “The precise details of how this principle is applied vary somewhat from policy to policy but the general principle entails estimating the fraction of total GHG emissions that are attributable to a particular sector (transport, housing, etc.) and then to the relevant target of action within each sector.”

 Also, please check the link here. The version linked (v4) threw a “DOI Not Found” error. For the purposes of my review, I used 10.6084/m9.figshare.13011533.v3 instead

Conclusions:

 “new rapid assessment tool (CRAFT), *Extended data*, Supplementary File 3 ( Symonds *et al.*, 2020c),”

 Please reference the supplemental link to the tool on first mention of CRAFT in the manuscript.

**Minor Typos/wording:**

Abstract:

 “including detailed policies” recommend replace with “and accompanying detailed policies” to better distinguish the roles of policy targets and policy instruments

 Suggest replacing “magnitude of impact… to reach” with “magnitude of impact… toward reaching”

Results

 In “on the detail of the policy implementation” replace “detail” with “details”

Discussion:

 Add “the” before “apparent NO2 effect”

 Punctuation is odd, here, in its separation of the subject “calculations” from the verb “represent”.

 “The calculations for the ‘Buildings upgraded’ policy ( *Up to 50% of buildings upgraded by 2025, 100% upgraded by 2050*), see *Extended data*, Supplementary Files 1 and 2 ( Symonds *et al.*, 2020a; Symonds *et al.*, 2020b), for full details, represent…”

 Recommend instead:

 “The calculations for the ‘Buildings upgraded’ policy ( *Up to 50% of buildings upgraded by 2025, 100% upgraded by 2050*; see *Extended data*, Supplementary Files 1 and 2 ( Symonds *et al.*, 2020a; Symonds *et al.*, 2020b) for full details) represent…”

CRAFT tool

 In the tab "Indoor Air Quality" cell J6 appears mislabeled. It should read Radon not PM2.5.

 Suggest changing wording in cell G6 to reference outdoor-source PM2.5 to better distinguish it from cell D6 and map with other supplemental material.

Is the work clearly and accurately presented and does it cite the current literature?

Yes

If applicable, is the statistical analysis and its interpretation appropriate?

Yes

Are all the source data underlying the results available to ensure full reproducibility?

Yes

Is the study design appropriate and is the work technically sound?

Yes

Are the conclusions drawn adequately supported by the results?

Yes

Are sufficient details of methods and analysis provided to allow replication by others?

Yes

Reviewer Expertise:

Integrated modelling of air pollution and associated health and economic impacts under climate change and climate policy.

I confirm that I have read this submission and believe that I have an appropriate level of expertise to confirm that it is of an acceptable scientific standard.

# Peer review: Reviewer response for version 2

**DOI:** 10.21956/wellcomeopenres.18616.r43953

**Published:** 2021-07-14

**Authors:** George D. Thurston

**Affiliations:** 1 Department of Environmental Medicine, New York University School of Medicine, New York City, NY, USA

Nothing to add.

Is the work clearly and accurately presented and does it cite the current literature?

Yes

If applicable, is the statistical analysis and its interpretation appropriate?

Partly

Are all the source data underlying the results available to ensure full reproducibility?

Yes

Is the study design appropriate and is the work technically sound?

Yes

Are the conclusions drawn adequately supported by the results?

Yes

Are sufficient details of methods and analysis provided to allow replication by others?

Yes

Reviewer Expertise:

air pollution exposure assessment and epidemiology

I confirm that I have read this submission and believe that I have an appropriate level of expertise to confirm that it is of an acceptable scientific standard.

# Peer review: Reviewer response for version 2

**DOI:** 10.21956/wellcomeopenres.18616.r43952

**Published:** 2021-05-19

**Authors:** Patrick L. Kinney

**Affiliations:** 1 Department of Environmental Health, Boston University School of Public Health, Boston, MA, USA

No further comments, I approve the article.

Is the work clearly and accurately presented and does it cite the current literature?

Yes

If applicable, is the statistical analysis and its interpretation appropriate?

Not applicable

Are all the source data underlying the results available to ensure full reproducibility?

Yes

Is the study design appropriate and is the work technically sound?

Yes

Are the conclusions drawn adequately supported by the results?

Yes

Are sufficient details of methods and analysis provided to allow replication by others?

Yes

Reviewer Expertise:

NA

I confirm that I have read this submission and believe that I have an appropriate level of expertise to confirm that it is of an acceptable scientific standard.

# Peer review: Reviewer response for version 1

**DOI:** 10.21956/wellcomeopenres.17972.r42550

**Published:** 2021-03-02

**Authors:** George D. Thurston

**Affiliations:** 1 Department of Environmental Medicine, New York University School of Medicine, New York City, NY, USA

Nations are doing poorly at achieving the climate forcing emission reductions needed to avoid a climate calamity. Even if the world’s countries follow through on present plans, their combined impacts would put us on a path to achieve only a 1 % reduction in global emissions by 2030, while climate scientists tell us that we need more than that each and every year. It is clear that more progress is needed, and action by individual cities are one pathway to jump start action, but motivation is needed.

 In “A tool for assessing the climate change mitigation and health impacts of environmental policies: the Cities Rapid Assessment Framework for Transformation (CRAFT)” Symonds *et al *provide a much needed city-level tool for the documentation of the potential local health benefits of climate mitigation measures, and their monetary valuations. The methods incorporated in the framework, while straightforward, are useful and consistent with present cost-benefit approaches.

 I do have some concerns with the specifics, however. The estimates provided lack confidence intervals, as acknowledged by the authors, but which would be important for decision-makers to consider. In addition, the NO _2_ mortality estimates (though not used in the final estimates) are not as confident as the PM2.5 estimate, as the causality of that relationship has weaker causal foundation. The original COMEAP Report that is referenced, it is stated that: “A majority view of the Committee was that approximately 25-55% of the unadjusted summary coefficient derived from the meta-analysis of single pollutant models (1.023 per 10 μg/m3 NO2) could be regarded as causally related to NO2. This gives a range of between 1.006 per 10 μg/m3 and 1.013 per 10 μg/m3”, not the 1.023 per 10 μg/m3 used in the analysis. A smaller NO _2_ estimate would be more defendable, but I would recommend dropping the NO _2_ estimates from the report.

Is the work clearly and accurately presented and does it cite the current literature?

Yes

If applicable, is the statistical analysis and its interpretation appropriate?

Partly

Are all the source data underlying the results available to ensure full reproducibility?

Yes

Is the study design appropriate and is the work technically sound?

Yes

Are the conclusions drawn adequately supported by the results?

Yes

Are sufficient details of methods and analysis provided to allow replication by others?

Yes

Reviewer Expertise:

air pollution exposure assessment and epidemiology

I confirm that I have read this submission and believe that I have an appropriate level of expertise to confirm that it is of an acceptable scientific standard, however I have significant reservations, as outlined above.

# Peer review: Reviewer response for version 1

**DOI:** 10.21956/wellcomeopenres.17972.r42483

**Published:** 2021-02-23

**Authors:** Patrick L. Kinney

**Affiliations:** 1 Department of Environmental Health, Boston University School of Public Health, Boston, MA, USA

This paper documents the structure and function of a new tool that has been developed to assist cities in weighing the climate and health benefits of various interventions. It is meant to be a rapid assessment tool that can be readily applied by non-scientists. I find this very solid and well supported. I did have a slight concern about the treatment of greenspace benefits, for which only a small subset of health benefit pathways are quantified. The authors fully acknowledge this in the discussion. However, I wonder if it's better to leave greenspace out of the example rather than to leave the impression that the benefits are so low. Alternatively, could include a sensitivity analysis using the exposure-response function from a recent meta analysis, such as Lancet Planet Health 2019;3: 469–77 ^1^. 

 Minor comment: second paragraph of page 12. First sentence. The parentheses seem mis-placed.

Is the work clearly and accurately presented and does it cite the current literature?

Yes

If applicable, is the statistical analysis and its interpretation appropriate?

Not applicable

Are all the source data underlying the results available to ensure full reproducibility?

Yes

Is the study design appropriate and is the work technically sound?

Yes

Are the conclusions drawn adequately supported by the results?

Yes

Are sufficient details of methods and analysis provided to allow replication by others?

Yes

Reviewer Expertise:

Health effects of air pollution and climate change. Health benefits of carbon mitigation strategies.

I confirm that I have read this submission and believe that I have an appropriate level of expertise to confirm that it is of an acceptable scientific standard.

## References

[1] : Green spaces and mortality: a systematic review and meta-analysis of cohort studies. *The Lancet Planetary Health* .2019;3(11) : 10.1016/S2542-5196(19)30215-3 e469-e477 10.1016/S2542-5196(19)30215-3 PMC687364131777338
